# Medical Opinion and Movement

**Published:** 1911-08-12

**Authors:** 


### Medical Opinion and Movement.

### Forster's Operation.

Resection of the posterior spinal nerve-roots for the relief of extreme pain in the corresponding parts has not been carried out extensively in this country, but some very interesting reports upon the operation and its effects are given by E. W. H. Groves, Esq., M.S., F.R.C.S., in the *Bristol Medico-Chirurgical Journal* for June 1911, pp. 105 to 109. The conditions in which he has performed the operation chiefly have been: for the lightning pains of locomotor ataxia; for the gastric crises of locomotor ataxia; for the relief of severe gastric pains other than those due to locomotor ataxia; and for the relief of extreme spasticity in connection with lesions of the pyramidal tracts. Mr. Groves candidly admits that in certain of these conditions the pains come back notwithstanding the operation, owing to the fact that they are of central rather than of peripheral origin. In these cases, particularly in association with the lightning pains of locomotor ataxia, the benefit likely to be derived from the operation depends to a considerable extent upon the absence of hysteria, but the operation seems to afford a most potent means of stopping the visceral crises of tabes dorsalis, and it will possibly prove of great value in this condition if applied to suitably selected cases, whilst it also cures the muscular spasm which is so extremely inconvenient in many cases of disease of the pyramidal tracts. Resection of the posterior nerve-root is a very severe operation, especially when it is performed on patients who are already weak and ill, but it is borne far better than might be expected.

### Artificial Thyroid Extract.

Thyroid extract is so very important a drug in the treatment of many different affections that it is essential that one should be sure of the activity of the preparation that is being employed. Nevertheless there is at present no authoritative standard for it nor any fixed method for its preparation, but a detailed account of a good process for its manufacture is given by Dr. Beebe in the *Pharmaceutical Journal*. It is small wonder that the price of some varieties of thyroid extract is very much less than that of others when one learns that at any rate some of that which is upon the market is not made from thyroid glands at all, but consists of varied proteids to which iodine has been added artificially. The profession needs to be warned of this, for otherwise, unless the source and the mode of preparation of the thyroid extract that may be employed are both known, it may be that the failure of the remedy to cure is not really failure of the thyroid extract at all, but the failure of an active substitute.

### X-ray Signs in Mastoid Disease.

Skiagraphic diagnosis of disease of the mastoid cells and antrum is necessarily open to much difficulty, owing to the varying opacity of the cranial bones and to the great opacity of the petrous bone in particular; nevertheless modern methods are making it less and less difficult to examine the cranium with the *x*-rays, and in a paper read before the College of Physicians of Philadelphia by Dr. Sydney Lange, stress is laid upon the value of skiagraphy in the recognition, and in the determination of the degree, of mastoiditis. Lange tabulates the pathological changes which may be noted upon the skiagram as follows: 1. Slight haziness of the cell-spaces or complete filling of them with something denser than air. These changes represent clinically otitis media, with slight mastoid involvement and the milder forms of mastoiditis. The something which fills the cells is in all probability serum. To show these slight changes the skiagram must be made with a soft tube and slightly under-exposed. 2. Loss of distinctness of cell walls with partial destruction of them. Clinically these changes are found in the more severe grades of mastoiditis, with pus formation and bone softening. 3. Complete loss of structure of the mastoid process, occurring either (*a*) in the form of localised abscess formation or (*b*), as is often seen in the sub-acute cases, in the form of extensive loss of structure, indicating advanced necrosis of the entire mastoid process. 4. Increased density of the mastoid process, with (*a*) partial or complete obliteration of cells and (*b*) with bone defects. This class represents the chronic sclerosed cases.

### Sulphur as Medicine.

Sulphur has once more come into vogue for the relief of various intestinal conditions, and, therefore, some researches upon the action of sulphur upon the alimentary canal by Frankl (*Archiv. für Experimentelle Pathologie und Pharmakologie*, June 1911, pp. 303-308) are of particular interest. As the result of a large amount of experimental work two conclusions stand out pre-eminently. The first of these is that there is no particular danger that the administration of sulphur will cause any unpleasant odour either of the breath or of the body generally, for Frankl has shown that he was uniformly unable to demonstrate the conversion of sulphur into sulphuretted hydrogen within the bowel; and the second is that the laxative action of sulphur depends, at least in part, on its oxidation to sulphurous acid, which, in addition to being a disinfectant, has a stimulating effect upon the intestinal mucous membrane, producing both hyperæmia and increased peristalsis.

### An Operation for Varicocele.

The supra-scrotal operation for varicocele, with intentional ligature of the spermatic artery, is advocated by Dr. E. S. Potter in the *International Journal of Surgery*. The author has performed this operation 214 times, and finds the results superior to the usual methods of ligature of the veins without occlusion of the artery. In sixty-seven cases which he has been able to follow up, no instance of atrophy of the testis has occurred; and the author believes that such a thing never happens from tying the spermatic artery. In about one-tenth of the cases there was a certain amount of slight septic infection—stitch abscess or the like. Neither hæmatocele nor phlebitis was encountered at all; and hydrocele followed in but three patients. When the veins are obliterated but the artery left patent, œdema and swelling of the testis not infrequently result, and persist until sufficient compensatory enlargement of some small vein left intact has time to take place; these complications are not to be feared if the artery itself is occluded as well as the veins. So convinced is this author of the importance of this factor in the operation that he proposes to tie the artery without touching the veins at all.

### Functional Albuminuria.

The most important points connected with this very common condition are summarised by Dr. R. Hutchison in a lecture which is published in the *Clinical Journal*. In view of its bearings on life insurance, choice of career, and so on, this condition is one about which everyone in practice is bound to be called upon some day for a pronouncement. Dr. Hutchison does not believe that true functional (or cyclical, orthostatic, postural, physiological, intermittent) albuminuria is of any serious significance; in other words, he does not regard it as the precursor of kidney lesions of a more serious nature. The main basis of distinction between this functional albuminuria and that due to definite renal disease rests on two facts. The first is that functional albuminuria is not present on first rising in the morning, but comes on after being up for an hour or two. The other is that granular casts are never present, though the hyaline variety may be. Another point of distinction is that acetic acid in the cold will often give a definite cloud with a functional case, but not in organic albuminuria; this is due to the presence of mucin or nuclein compounds. Calcium lactate, which has been suggested for so many different disorders the last few years, has been tried by Dr. Hutchison and found wanting. The line he adopts is to attend to the general health and to let the albuminuria look after itself.

### Milk and Disease.

Dr. Collingridge, medical officer of health for the City of London, has published a summary of the results obtained from testing forty-two samples of milk by injections into guinea-pigs. Apparently in no case tuberculosis resulted. This is said to be the first time that all samples have been free from evidence of the presence of the tubercle bacillus. Of the forty-two, however, in only twenty-two were the results of injection in the guinea-pigs negative. In the remaining twenty either acute local inflammation and abscess resulted or rapid death. This is taken as evidence of dirty milk and evidence of either an unsatisfactory state at the farm or carelessness in transit. If the results are not open to criticism and can be taken as evidence of the frequency of pathogenic micro-organisms in milk, there must be plenty of room for improvement in the circumstances amidst which milk is obtained and distributed for the use of the public. Not long ago a daily paper quoted the remark of a resentful farmer, a member of a county council before which suggestions as to care of milk had been laid. The farmer remarked that he " did not use tooth-brushes for his cows." No one wishes to hamper our English farmer. He has plenty of troubles. Care as to cleanliness regarding milk is, however, far removed from faddism.

### Rapid Death in Childhood.

Among the recorded cases of death noticed in the daily papers, said to be due to the heat, was the mention of a boy aged ten years, who is said to have died in a few hours of acute congestion of the lungs. This was probably an example of a remarkable and obscure form of death in children, in which asphyxia with some rise of temperature are the main symptoms. A case has come under our notice, in which a boy died in the course of a few hours of acute dyspnœa, where after death great œdema of the lungs was present. Serous fluid filled the bronchial tubes and poured out from the cut surfaces of the lungs. In this case there were recent vegetations on the aortic valve. This suggested that a blood infection had been present for a few days and that the œdema of the lungs caused a rapid termination. Many similar cases have been noticed where children are collected together in resident schools and have been classed under " rapidly fatal institutional diseases." Dr. Charles Macalister has given notes of several cases which occurred in an industrial school of which he was medical officer. One or two illustrations may be given. During an entertainment for the boys in the school, one was noticed to be troubled with an irritating cough. Some sweets were given to him, which he sucked without relief. The cough continuing, he went early to bed. Half an hour later the matron was passing through the dormitory, noticed that he was very still, and found him to be quite dead. As she turned his head some fluid escaped from the mouth. In another case death occurred about eight hours after the onset of signs of indisposition. In this case the temperature was 101.6°, there was dyspnœa, and the boy was intensely cyanosed. At the autopsy the lungs were much congested and dirty fluid exuded from the cut surfaces. Apparently the cause of death in these cases is asphyxia, due to acute œdema of the lungs.

### Tincture of Iodine in Erysipelas.

The uses of tincture of iodine as an antiseptic are daily becoming better recognised. Dr. Ferrari Mario now describes (in the *Gaz. degli ospedali et delle cliniche*) its advantages in cases of facial erysipelas. Method of treatment: (1) The zone of invasion of the erysipelatous infection must be painted with a sterilised tampon of cotton-wool impregnated with tincture of iodine; (2) next the infected area must be painted in a similar way with a second tampon; (3) the infected area and the part immediately surrounding it are then dressed with sterilised wool. This dressing is very important, in order to prevent scratching and secondary infection. The iodine solution should be freshly made and of a strength of 10 to 12 per cent. In the beginning the author made two applications, morning and evening, though he found later that even better effects were obtained by administering five or six less thorough paintings through the day. He has used this method with excellent results in thirty-eight cases of erysipelas in different parts of the body.

### A Case of Coffee-Poisoning.

Dr. Bardet recently reported to the Société de Therapeutique a case of acute poisoning from coffee drinking. The amount of coffee taken by the patient corresponded at least to 0.70 gram of caffeine. The patient, a chronic dyspeptic with hepatic insufficiency, had always been susceptible to coffee, especially when taken in the evening, and because of this failing had substituted caffeine-free coffee for the ordinary variety. Unfortunately for him, the night of the accident he had by a mistake been served with ordinary coffee, which he had taken with milk. His symptoms then were as follows: Very rapid heart-beat and pulse-rate; painful, scanty, and very infrequent micturition; considerable excitement, followed by profound prostration, the whole lasting for three days. The author, as the result of this observation, states that nervous dyspeptics, especially those with a tendency to become excited, should be very sparing in the use of coffee. Caffeine-free coffee, though perhaps less palatable, should be of great service in such cases.

### Diet in Nasal Affections.

A number of chronic affections of the upper respiratory passages, and in particular of the nose and pharynx, are dependent on a humoral disturbance rather than on a local lesion or infection. Cornet showed that certain chronic coryzas result from latent Bright's disease, as evidenced by traces of albumen in the urine, whereas neither œdema nor other symptoms of the malady can be found. Treatment by a lacto-vegetarian diet free from chlorides quickly caused a cessation of the nasal discharge. More recently Meyjes of Amsterdam has proved that chronic congestion of the nasal mucous membranes coincides with hyperacidity of the urine and an excessive meat diet. Max Senator, after reviewing these facts in the *Medizin. Klin.*, states that when patients come to consult him he always forbids the use of spiced, acid, and salty foods, condiments such as vinegar, mustard, and pepper, onions and cucumbers, which seem to have an unfavourable effect on the respiratory passages. The nitrogenous elements should be reduced, as also alcoholic drinks. Liquids should be taken at the temperature of the room, as cold and hot drinks tend to congest the mucous membranes. These directions are to be observed both before and after local treatment, and naturally they are only meant to supplement any dietary that has already been fixed because of some existing general disease.

### Trichloracetic Acid in Dermatology.

In a recent number of the *Münchener Medizinische Wochenschrift*, Dr. G. Knauer recommends the use of trichloracetic acid in all cases which are at present treated by carbonic acid snow. He claims that the drug is equally efficient, less costly, and much simpler to use than the snow, with the proviso that none of the acid be allowed to touch healthy tissues. To prevent this a ring of collodion should be painted round the affected area. After liquefying the acid with a few drops of water, the solution is applied with glass rods varying in size according to the area of tissues to be treated. The resulting cauterisation is superficial unless the drug has been rubbed in. A second application is rarely necessary, and in any case should not be given until the scab of the first has dropped off. After cauterisation the area treated shows up snow-white, with a surrounding ring of hyperæmia. In a few hours this area turns brown, but no vesicles are formed. The scab is loose in eight or ten days. The ultimate appearance of the part is good and approaches that seen after treatment with snow. The applications are said to be practically painless. The author finds the snow more useful than the drug when large areas are to be dealt with.

### Gastric Syphilis.

Syphilitic disease of the stomach is not generally recognised in the text-books, and indeed it is usually taught that there is no syphilitic lesion of the alimentary canal between the pharynx above and the rectum below. The fact that gastric symptoms, simulating gastric ulcer, may be cured entirely by anti-syphilitic remedies even after the more usual treatment for gastritis or gastric ulcer has failed after prolonged trial, would seem to indicate the possibility of syphilis of the stomach being a real pathological entity. A case in point has recently been recorded at considerable length by Béclère and Bensaude in the Bulletins et Mémoires de la Société Médicale des Hôpitaux de Paris, 1911, p. 680. The gist of the case is that the patient was a man aged fifty-four, who had suffered from severe epigastric pain extending over eleven years, and a more recent loss of weight of 20 kilogrammes; examination with bismuth and the *x*-rays showed a definite degree of hour-glass contraction; and no remedies brought about any material relief until, when surgical assistance was being considered, it was decided to give biniodide of mercury hypodermically a trial first. Two centigrammes were given by the intra-muscular method every two days and the patient experienced rapid relief, increased in weight, lost his pains, became able to digest almost anything; and whereas before this treatment was adopted he could scarcely walk, he is now able to go about vigorously.

